# Experimental investigation of a four-qubit linear-optical quantum logic circuit

**DOI:** 10.1038/srep33475

**Published:** 2016-09-20

**Authors:** R. Stárek, M. Mičuda, M. Miková, I. Straka, M. Dušek, M. Ježek, J. Fiurášek

**Affiliations:** 1 Department of Optics, Palacký University, 17. listopadu 1192/12, 771 46 Olomouc, Czech Republic

## Abstract

We experimentally demonstrate and characterize a four-qubit linear-optical quantum logic circuit. Our robust and versatile scheme exploits encoding of two qubits into polarization and path degrees of single photons and involves two crossed inherently stable interferometers. This approach allows us to design a complex quantum logic circuit that combines a genuine four-qubit C^3^Z gate and several two-qubit and single-qubit gates. The C^3^Z gate introduces a sign flip if and only if all four qubits are in the computational state |1〉. We verify high-fidelity performance of this central four-qubit gate using Hofmann bounds on quantum gate fidelity and Monte Carlo fidelity sampling. We also experimentally demonstrate that the quantum logic circuit can generate genuine multipartite entanglement and we certify the entanglement with the use of suitably tailored entanglement witnesses.

Since the seminal proposal by Knill, Laflamme and Milburn^1^ of an all-optical scalable quantum computing architecture, the field of linear optical quantum computing has experienced immense growth and expansion^2^,^3^. Various elementary quantum gates for qubits encoded into states of single photons have been demonstrated^4^,^5^,^6^,^7^,^8^,^9^,^10^,^11^,^12^,^13^,^14^,^15^,^16^, the optical quantum logic circuits have been miniaturized and integrated on a photonic chip^17^,^18^,^19^,^20^, and alternative more efficient approaches to all-optical quantum computing such as utilization of photonic cluster states^21^,^22^ have been developed. The number of optical photons which can be simultaneously generated and coherently processed has also increased in time^23^,^24^,^25^ up to the very recent record of 10 photons^26^. However, further scaling is largely prevented by the probabilistic nature of current sources of photons based on spontaneous parametric down-conversion, and deterministic single-photon sources of sufficient quality are not yet fully available despite recent significant progresses^27^,^28^,^29^.

Instead of increasing the number of photons one could exploit multiple degrees of freedom to encode several qubits in state of a single photon^30^. Although the total number of optical modes then increases exponentially with the number of qubits, this approach may nevertheless prove very useful for development of specific small-scale optical quantum circuits that can find applications, e.g., in nodes of optical quantum communication networks. Important examples of the simultaneous exploitation of several degrees of freedom of single photons for encoding and processing quantum information include generation of hyper-entangled photon pairs^31^, superdense quantum teleportation^32^, design of certain linear optical quantum logic gates^14^, and implementation of random quantum walks^33^,^34^,^35^.

In this work, we exploit the polarization and path degrees of freedom to encode two qubits into a single photon and construct a two-photon four-qubit linear optical quantum logic circuit, which represents an important step beyond the previous implementations of two-4,5,6,7,8,9,10,11,13 and three-qubit^14^,^15^,^16^ linear optical quantum gates. The implemented quantum logic circuit is illustrated in Fig. 1(a). It combines several two-qubit quantum controlled-rotation gates, single-qubit gates, and a four-qubit generalized controlled-Z gate, which flips the sign of the state only if all four qubits are in the computational state |1〉,


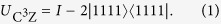


Here *I* denotes the identity operator on Hilbert space of four qubits. In our implementation, the qubits 1 and 2 are encoded into the polarization and path of the signal photon, respectively, while qubits 3 and 4 are similarly encoded into polarization and path of the idler photon. The two-qubit controlled-rotation gates applied to polarization and path qubits supported by the same photon can be implemented deterministically, while the core four-qubit C^3^Z gate is probabilistic, with theoretical success probability of 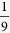.

The four-qubit quantum logic circuit is a complex device, and its complete experimental characterization would require determination of 2^16^−1 = 65535 parameters. Here we employ Hofmann fidelity bounds^15^,^36^ and Monte Carlo sampling techniques^37^,^38^ to efficiently characterize the performance of the quantum logic circuit. Our scheme provides a suitable platform for testing and illustrating the usefulness of these methods, which can also serve for efficient evaluation of other kinds of multi-qubit quantum gates. We find that the fidelity 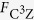 of the four-qubit C^3^Z gate lies in the interval 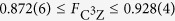 and the Monte Carlo sampling provides an explicit fidelity estimate 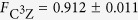. We show that our device can generate four-qubit GHZ-type entangled state whose fidelity with the ideal state and purity exceed 90%. Moreover, using suitable entanglement witnesses we verify that the generated state exhibits genuine four-partite entanglement. The developed scheme combining two crossed inherently stable optical interferometers provides a promising configuration for design of even more complex linear-optical quantum information processing devices.

## Results

### Experimental setup

**Figure 1:**
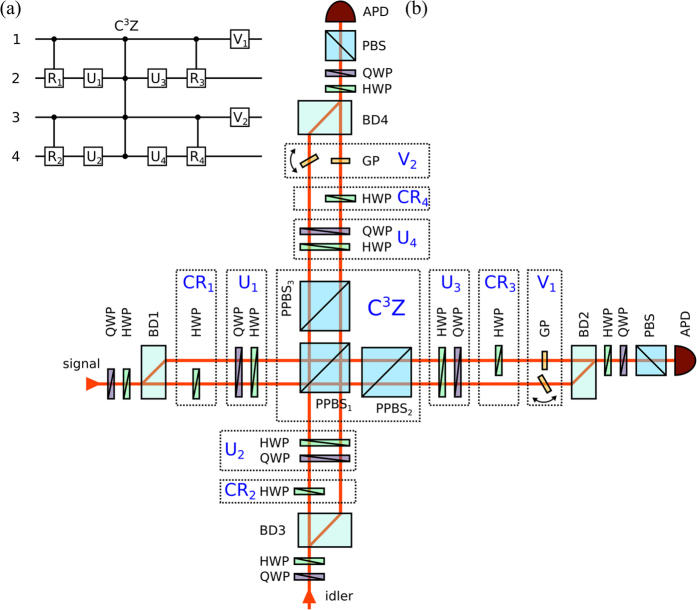
(**a**) Scheme of the implemented quantum logic circuit. (**b**) Experimental setup. The central four-qubit C^3^Z gate is implemented by two-photon interference on a partially polarizing beam splitter PPBS_1_ followed by two additional PPBSs which serve as partial polarization filters. Notice that only the left and lower beams overlap and interfere on PPBS_1_. Single-qubit unitary gates *U*_*j*_ are implemented by a sequence of a rotated half-wave plate HWP and quarter-wave plate QWP which address both paths in a Mach-Zehnder interferometer formed by two calcite beam displacers BD. The two-qubit controlled rotation gates CR_*j*_ are realized by a rotated HWP which is inserted only in one arm of the interferometer. Single-qubit phase gates *V*_*k*_ are achieved by tilting a glass plate GP inserted in one of the interferometer arms. The output states of photons are analyzed and detected with the help of wave plates, polarizing beam splitters PBS and avalanche photodiodes APD.

In our experiment, a pair of time correlated orthogonally polarized photons at wavelength 810 nm is generated by spontaneous parametric down-conversion in a type-II nonlinear crystal pumped by a cw laser diode. The signal and idler photons are spatially separated at a polarizing beam splitter and injected into the two input ports of the interferometric setup which is depicted in Fig. 1(b) and consists of two crossed Mach-Zehnder interferometers formed by calcite beam displacers^14^,^15^. Each photon from the pair carries two qubits encoded into its polarization and path degrees of freedom. The horizontal and vertical polarizations of the photon represent the computational states |0〉 and |1〉 of the polarization qubit, respectively. Similarly, propagation in the displaced and straight arm of a Mach-Zehnder interferometer represents computational states |0〉 and |1〉 of the path qubit.

Path qubits are initially prepared in polarization encoding using combination of half-wave plate (HWP) and a quarter-wave plate (QWP) and subsequently converted into path encoding using a polarizing beam displacer (BD). The polarization-to-path conversion produces path-polarization entangled states. We can disentangle the state using a HWP that addresses only the straight arm of the interferometer. The action of this HWP rotated at 45° can be regarded as a quantum CNOT gate acting on the spatial control and polarization target qubits. Arbitrary polarization qubits can be prepared by an additional pair of HWP and QWP plates affecting both arms of the interferometer. The output state analysis blocks are counterparts of the preparation blocks, operating in a similar way and consisting of the same elements up to the final polarizing beam splitters and avalanche photodiodes. We can project the output photons onto an arbitrary product four-qubit state, and with suitable rotations of the HWPs addressing only the displaced arms of the interferometers we can also perform projections onto products of various two-qubit entangled states. The electronic signal from the avalanche photodiodes is processed by coincidence logic, and the number of coincidences detected during the measurement time is stored in a computer.

The core part of our linear optical quantum logic circuit consists of two-photon interference on the central partially polarizing beam splitter PPBS_1_. Implementation of the four-qubit C^3^Z gate requires that the two photons interfere only if their path qubits are both in the state |1〉, i.e. if they both propagate in the straight interferometer arms. To avoid interference of photons propagating in the displaced arms of the interferometers, we introduce different transverse separations of the interferometer arms for the two interferometers. Specifically, the beams are separated by 4 mm and 6 mm in the signal and idler interferometers, respectively, and the optical beams have diameter of 2 mm. The beams are adjusted such that the photons propagating in the straight arms perfectly overlap, while the photons propagating in the displaced arms are mutually transversally shifted and do not overlap on PPBS_1_. The nominal reflectances of PPBS_1_ read *R*_*H*_ = 0 and *R*_*V*_ = 2/3 for horizontally and vertically polarized photons, respectively. The other two partially polarizing beam splitters have inverted reflectances *R*_*H*_ = 2/3 and *R*_*V*_ = 0 so that the combinations of PPBS_1_ & PPBS_2_ and PPBS_1_ & PPBS_3_ form polarization insensitive gray filters with overall transmittance 1/3.

The above described optical design ensures that the conditional *π*-phase shift induced by the two-photon interference on PPBS_1_ occurs if and only if all four qubits are in the logical state |1〉. The physical principle of the conditional phase shift is the same as for the two-qubit linear optical quantum CZ gate demonstrated in Refs 7, 8, 9. The gate operates in the coincidence basis, its success is heralded by detection of a single photon in each output port of the gate, and the theoretical success probability of the four-qubit C^3^Z gate is 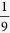. The Mach-Zehnder interferometers formed by beam displacers are inherently stable and this stability is preserved even for the present configuration with two crossed interferometers. We observed that the whole setup is passively stable on the time scale of hours, which enables detailed and comprehensive characterization of the implemented quantum logic circuit.

### Experimental characterization of the four-qubit quantum C^3^Z gate

**Figure 2:**
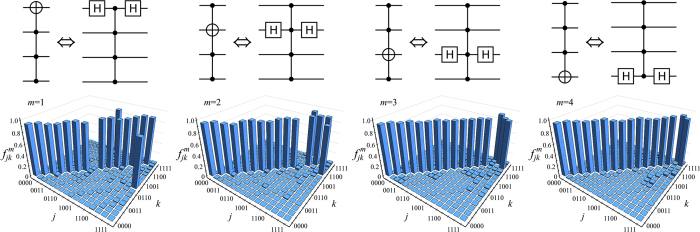
Experimentally determined truth tables of the four-qubit quantum Toffoli gates. The quantum logic circuits in each case indicate which qubit is the target qubit, and they also illustrate that each Toffoli gate is equivalent to a suitable combination of the C^3^Z gate and two single-qubit Hadamard gates.

As illustrated in Fig. 2, the four-qubit C^3^Z gate, which is a central part of our linear optical quantum logic circuit, is equivalent to a four-qubit Toffoli gate up to local single-qubit Hadamard transforms on the target qubit. For each of the four choices of the target qubit we have measured the truth table of the resulting Toffoli gate, which illustrates its performance in the computational basis. The Hadamard transforms on the target qubit were implemented with the use of wave plates, which can be equivalently seen as probing the four-qubit CZ gate with a product state consisting of three control qubits prepared in the computational basis states |0〉, |1〉 and one target qubit prepared in the superposition basis state 
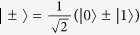
. At the output of the four-qubit C^3^Z gate, the three control qubits are measured in the computational basis while the target qubit is measured in the superposition basis. The experimentally determined truth tables are shown in Fig. 2. The truth tables of all four Toffoli gates clearly show the expected bit flip of the target qubit conditional on all control qubits being prepared in the computational basis state |1〉.

We now specify in more detail the quantities plotted in Fig. 2. Let 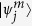 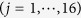 denote the *j*th input four-qubit state when *m*th qubit is the target qubit. The corresponding output state is given by 
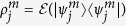
 where 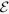 is the four-qubit quantum operation actually implemented by our setup. Note that, due to various experimental imperfections, 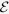 can be a general trace-decreasing completely positive map. The output density matrices 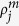 are not normalized, and 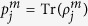 is the probability of success of the gate for a given input state 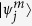. The truth tables depicted in Fig. 2 contain plots of the matrices 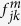 of normalized overlaps of the actual output state 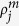 with the ideal output states 
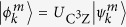
 produced by the perfect gate,


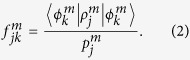


In particular, 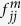 represents the output-state fidelity for input 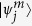.

In the experiment, we measure the number of two-photon coincidences 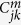 for all combinations of input state 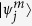 and output projection onto 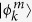. The measurement time is the same for all settings and set to 10 s. The normalized overlaps (2) are then estimated from the experimental data according to^15^


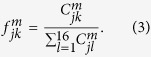


**Figure 3:**
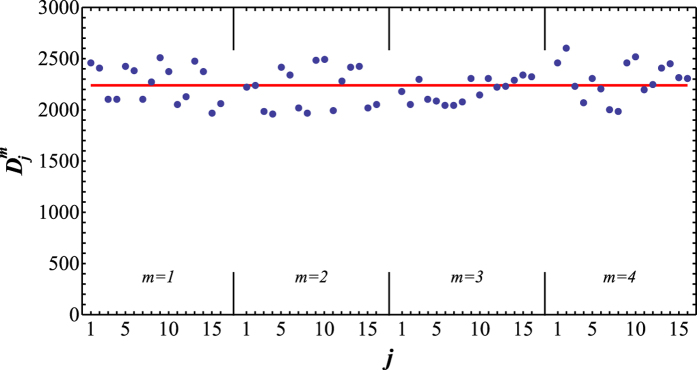
Total number of detected two-photon coincidences 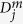 is plotted for the 64 input states 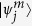 which were utilized in determination of the truth tables of the four-qubit Toffoli gates in Fig. 2.

For perfect albeit probabilistic implementation of a unitary gate, the success probabilities 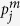 should be constant and independent on the input state. To assess the behaviour of 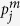 for our implementation, we plot in Fig. 3 the total number of detected two-photon coincidences 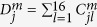 for each of the 64 considered input states 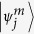. The variations of 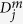 visible in the figure are partly caused by the Poissonian fluctuations of the number of emitted photon pairs. To compare the observed behaviour of 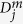 with Poissonian statistics, we use the data plotted in Fig. 3 to estimate the variance 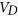 of 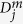 and compare it with the variance of a Poissonian distribution with the same mean number of coincidences 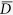. We obtain 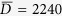 and *V*_*D*_ = 30338. This corresponds to relative fluctuations of 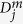 of 8% while the corresponding relative Poissonian fluctuations would be about 2% (one relative standard deviation). This indicates that due to the various technical imperfections our implementation of the C^3^Z gate introduces slight dependence of 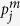 on the input state.

The data contained in the truth tables plotted in Fig. 2 can be used to derive a lower and upper bounds on the quantum process fidelity^39^,^40^ of the four-qubit C^3^Z gate,


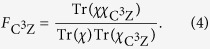


Here *χ* and 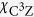 denote the Choi matrices^41^,^42^ of the actually implemented and the ideal C^3^Z gate, respectively. It holds that 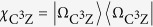, where


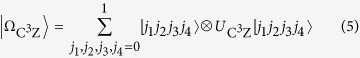


is a maximally entangled state of 8 qubits. For each choice of the target qubit *m* in Fig. 2, we define a weighted average state fidelity *F*_*m*_ as


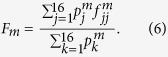


The four average state fidelities *F*_*m*_ determined from the truth tables of the four-qubit Toffoli gates plotted in Fig. 2 yield the following generalized Hofmann lower and upper bounds^15^,^36^ on the fidelity of the C^3^Z gate,


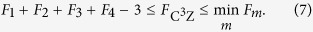


The experimentally determined average state fidelities read *F*_1_ = 0.943(1), *F*_2_ = 0.952(1), *F*_3_ = 0.944(1), and *F*_4_ = 0.955(1), which documents the very good performance of the gate. The statistical uncertainties of the fidelity estimates were obtained by error propagation assuming Poissonian statistics of the measured coincidence counts. If we insert these data into Eq. (7), we get 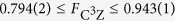. An experimentally appealing property of these fidelity bounds is that they can be determined by measuring fidelities of multiqubit output product states obtained from input product states. Therefore, neither preparation of entangled states nor measurements in an entangled basis are required. However, the resulting lower bound on 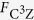 is rather loose.

To obtain a better and tighter lower bound on 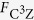 we have experimentally determined the original Hofmann fidelity bound^36^, which is given by average state fidelities 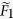 and 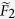 for two mutually unbiased bases. In particular, it holds that


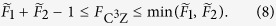


In our experiment, we construct the two mutually unbiased bases by preparing the polarization and path qubits of one photon in the computational basis states |0〉, |1〉 and the polarization and path qubits of the other photon in the superposition basis states |+〉, |−〉. The average state fidelities 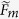 are defined similarly as *F*_*m*_. However, the determination of the corresponding output state fidelities 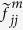 now requires measurements in entangled basis, since the C^3^Z gate maps some of the input states onto entangled output states. For instance,


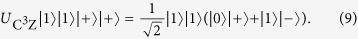


**Figure 4:**
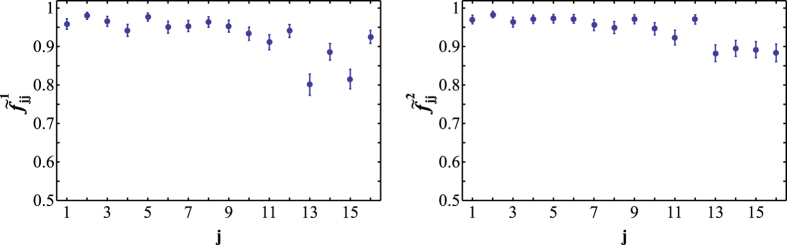
Experimental output state fidelities 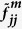 for input states forming two mutually unbiased bases. Each input state is a product state with two qubits prepared in the computational basis states and the other two qubits prepared in the superposition states |±〉.

Fortunately, the output state fidelities 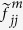 can be directly measured with our quantum logic circuit, because we can set the two controlled-rotation gates CR_3_ and CR_4_ in Fig. 1 to CNOT gates and perform measurements in the basis of maximally entangled Bell states. The experimentally determined output state fidelities 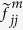 are plotted in Fig. 4 and the resulting average state fidelities read 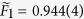 and 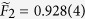. Consequently, we obtain the following bounds on the fidelity of the C^3^Z gate,


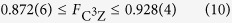


With a rather small number of measurements we have thus successfully confirmed the high-quality performance of our multiqubit quantum logic circuit and we have constrained the fidelity of the four-qubit quantum controlled-Z gate into a narrow interval.

### Preparation of four-qubit entangled state

We next investigate the ability of the C^3^Z gate to generate genuine multipartite entangled states from input product states. Specifically, we consider the input state |++++〉 which is transformed by the C^3^Z gate into an entangled state that belongs to the family of four-qubit GHZ states,


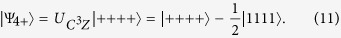


**Figure 5:**
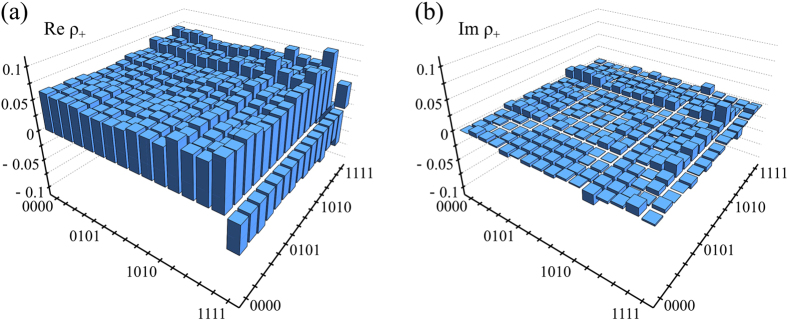
Real (**a**) and imaginary (**b**) part of the density matrix *ρ*_+_ of an entangled four-qubit state which was generated by the quantum C^3^Z gate from a product state |++++〉. The density matrix was reconstructed from the experimental data using the Maximum Likelihood Estimation procedure.

We have performed a tomographically overcomplete set of measurements on the generated four-qubit state and we have reconstructed its density matrix *ρ*_+_ from the experimental data using the Maximum Likelihood estimation procedure^43^,^44^. The resulting density matrix is plotted in Fig. 5. We can characterize the generated entangled state by its purity 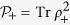 and fidelity 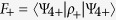. Using the experimentally determined *ρ*_+_ we obtain *F*_+_ = 0.942(2) and 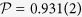 which demonstrates high quality of the generated state.

In order to certify that the experimentally prepared four-qubit state exhibits genuine multipartite entanglement, we utilize a suitable entanglement witness^45^,^46^,^47^. We recall that a multipartite quantum state exhibits a genuine multipartite entanglement if it cannot be written as a mixture of biseparable states. Consider first a maximally entangled GHZ state


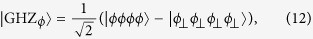


where 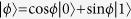 and 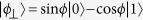 denote two orthogonal single-qubit states. An optimal projector entanglement witness^48^,^49^ for the state (12) is given by


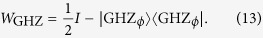


A genuine four-partite entanglement of a state *ρ* is certified when 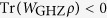, i.e. when the fidelity of the state with the maximally entangled GHZ state (12) exceeds 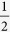. The overlap


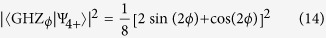


is maximized for 
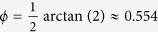
, and at this point 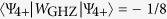. This shows that the standard GHZ witness (13) is capable to detect multipartite entanglement of the four-qubit state 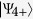. For the experimentally determined state *ρ*_+_ we find that the witness is minimized at 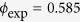, in excellent agreement with theoretical expectations. At this optimal point we get 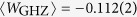 which confirms that the experimentally generated state exhibits genuine multipartite entanglement.

To complete our analysis we also present two alternative constructions of witnesses which can detect multipartite entanglement of the state 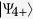. Our first construction is based on the observation that the four-qubit state 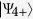 can be transformed onto the canonical GHZ state 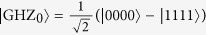 by local single-qubit operations. In particular, we have


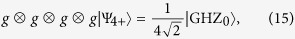


where


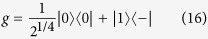


denotes a single-qubit quantum filter. Since local single-qubit quantum filters map biseparable states onto biseparable states, a filtered state 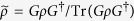, where 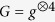, exhibits genuine multipartite entanglement only if the original state *ρ* also exhibits genuine multipartite entanglement. Starting from the optimal witness (13) for the canonical GHZ state 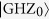, and considering its application to the filtered state 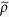, we arrive at the following witness for the original state *ρ*,


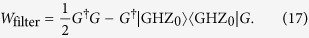


For the experimentally generated four-qubit state *ρ*_+_ we get 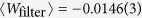, while for the ideal pure state (11) one has 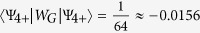. In our second approach we utilize a projector witness of the form


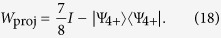


The proof that this is an optimal projector witness for the state 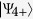 is provided in the Methods section. For the experimentally reconstructed state we obtain 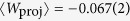.

A comparison of the three above discussed entanglement witnesses is provided in Table 1. Besides the mean values of the three witnesses, the table also shows the significance of the entanglement test^50^ defined as 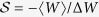, where Δ*W* is the standard deviation quantifying statistical uncertainty of 〈*W*〉. Moreover, the table also displays the maximum tolerable fraction of white noise *p*_max_ for which the witness still detects entanglement of a mixed state 
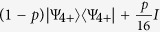
. We can see that *W*_GHZ_ is the optimal witness as it achieves the highest significance 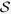 and also it can tolerate more white noise than the other two witnesses.

### Monte-Carlo sampling of quantum gate fidelity

The four-qubit quantum C^3^Z gate represents an interesting nontrivial device for testing techniques devised for efficient characterization of multiqubit quantum operations^37^,^38^,^51^,^52^,^53^,^54^,^55^,^56^,^57^. The Hofmann bound^36^ utilized in the previous part of our work can be considered as an example of such efficient partial characterization technique, but it should be noted that the number of measurement settings required for determination of the Hofmann bounds still scales exponentially with the number of qubits *N*. This exponential bottleneck can be avoided by the Monte Carlo sampling of quantum gate fidelity^37^,^38^,^51^,^52^. The main feature of this technique is that the number of measurement settings depends on the required uncertainty of the fidelity estimate but not on the dimension of the Hilbert space.

Here we apply the Monte Carlo sampling to determine the fidelity of the four-qubit quantum C^3^Z gate given by Eq. (4). The first step is to express the quantum process matrix 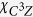 of the ideal C^3^Z gate as a linear combination of 8-fold tensor products of single-qubit Pauli operators *σ*_*X*_, *σ*_*Y*_, *σ*_*Z*_ and *σ*_0_ = *I*. We find that the expansion contains altogether 1936 different tensor products,


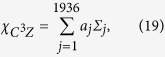


where 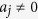 are real constants,


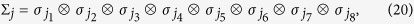



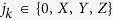
, and each parameter *j* labels a specific 8-fold tensor product (20). Since in our experiment we sequentially probe the quantum gate by various input states, we rewrite the expansion (19) as a linear combination of projectors onto pure product states. For this purpose, we express each of the three Pauli matrices *σ*_*X*_, *σ*_*Y*_ and *σ*_*Z*_ as a difference of projectors onto their +1 and −1 eigenstates and, similarly, we explicitly write 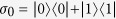. After some algebra, we arrive at the expansion


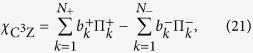


where 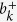 and 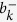 are positive coefficients,


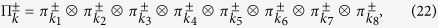


and each of the operators 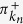 and 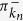 is a projector onto one of the eigenstates of *σ*_*X*_, *σ*_*Y*_ or *σ*_*Z*_. The total number of terms in the expansion (21) reads *N*_+_ = 22416 and *N*_*−*_ = 22400.

As a final preparatory step we introduce two probability distributions


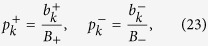


where


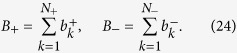


The Monte Carlo sampling proceeds as follows. We randomly generate a list of *M*_+_ labels *c*_*m*_ in the range [1, *N*_+_] drawn from a distribution 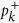, and we also generate a list of *M*_−_ labels *d*_*m*_ in the range [1, *N*_−_] drawn from the distribution 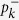. Next, we experimentally determine the mean values of the randomly chosen projectors 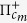 and 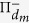,


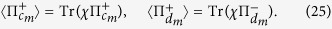


Practically, each of these terms can be measured by preparing a suitable four-qubit input product state and by performing a projection onto a suitable four-qubit product state at the output. Since the investigated optical quantum gate is probabilistic, we also need to carry out a reference measurement to determine the normalization factor 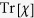. Generally, this normalization factor could be also estimated by Monte Carlo sampling. However, for the considered four-qubit gate it is possible to obtain the required reference by performing a complete measurement in the computational basis, which involves *M*_0_ = 2^8^ measurement settings. The gate fidelity is then estimated according to the formula


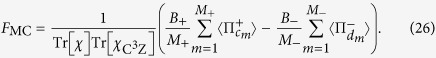


To assess the systematic error of gate fidelity estimation due to the finite number of samples *M*_+_ and *M*−, we assume perfect gate implementation, 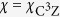, and neglect the statistical uncertainty of 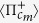 and 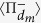. The systematic error of fidelity estimation due to finite number of samples can then be expressed as


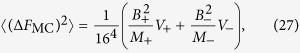


where


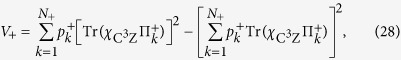


and 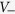 is defined similarly. For a fixed total number of samples 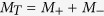 we can minimize the systematic error (27) by optimizing the number of samples *M*_+_ and *M*_−_. This yields


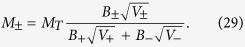


On inserting these optimal values back into Eq. (27) we obtain


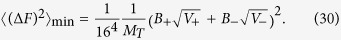


Numerically, we get


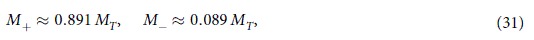


and


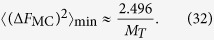


This indicates that the optimal sampling strategy is strongly unbalanced, with almost 90% of samples used to estimate the positive terms in the expansion (21) and only about 10% of samples is allocated to estimate the negative terms in that expansion. Formula (32) provides an explicit quantification of the systematic error of the Monte Carlo sampling procedure. To reduce the sampling error below 1% (as quantified by one standard deviation 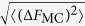), at least 2.5 × 10^4^ samples are required, which is comparable with the total number of terms *N*_+_ and *N*_−_ in the expansion (21).

**Figure 6:**
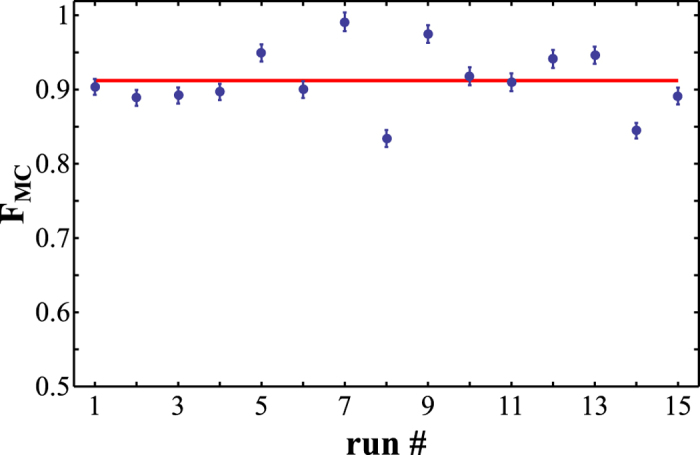
Monte Carlo estimates *F*_MC_ of fidelity of the experimentally implemented C^3^Z gate. Each estimate was obtained from *M*_*T*_ = 1100 samples and the whole sampling procedure was independently repeated 15 times to obtain an ensemble of fidelities. The error bars indicate statistical errors due to finite number of two-photon coincidence counts.

We have experimentally probed the performance of the Monte Carlo sampling procedure for our linear optical four-qubit quantum gate. We have generated the random list of *M*_*T*_ = 1100 measurement settings, measured the number of coincidences for a fixed time interval for all these settings, and we have also performed the measurements in the computational basis required for normalization. From these data we have determined the mean values (25) and obtained an estimate of the gate fidelity *F*_MC_. We have repeated this procedure 15 times and the resulting fidelity estimates are plotted in Fig. 6. We characterize the ensemble of fidelity estimates by its mean 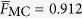 and standard deviation Δ*F*_MC_ = 0.042. This is consistent with the systematic error 0.048 predicted by formula (32) and the statistical error 0.011 due to Poissonian statistics of the coincidence events. The mean fidelity 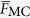 is in excellent agreement with the lower and upper Hofmann bounds (10). The uncertainty of 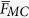 can be estimated as 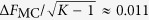, where *K* = 15 is the number of repetitions of the Monte Carlo sampling procedure.

We used the 16500 measured coincidences to reconstruct the implemented quantum process using the Maximum Likelihood estimation procedure. Since the data are tomographically incomplete, we have used 20 random operators at the beginning of the iterative reconstruction algorithm to check that the reconstruction always yields the same quantum process fidelity 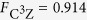 which lies inside the previously estimated boundaries.

## Discussion

In summary, we have experimentally demonstrated and characterized a four-qubit optical quantum logic circuit whose core is formed by a four-qubit C^3^Z gate. Our scheme exploits encoding of two qubits into polarization and path degrees of single photons and involves two crossed inherently stable interferometers. Our setup is passively stable on the time scale of hours which provided sufficient time for a comprehensive experimental characterization of the optical quantum logic circuit. We have verified high-fidelity performance of the central four-qubit C^3^Z gate and we have demonstrated that it can generate genuine multipartite entanglement. Our work illustrates that Monte Carlo sampling and Hofmann fidelity bounds are useful methods of characterization of complex multi-qubit quantum devices. The applicability of these methods is rather universal, and they can be used to efficiently characterize quantum logic gates and circuits implemented on various physical systems.

The implemented scheme combining two crossed interferometers is very flexible and with suitable modifications it may enable also realization of other quantum operations such as creation of superposition of unknown photonic quantum states and quantum Fredkin gate^58^. Moreover, by using additional calcite beam displacers, one could increase the number of paths in each interferometer which would enable experiments with multivalued quantum logic circuits where path degree of each photon supports a qudit instead of a qubit.

## Methods

### Optimal Projector witness for |Ψ_4+_〉

Here we construct the optimal projector witness


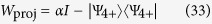


for the state 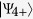 defined in Eq. (11). To optimize the witness (33), the coefficient *α* should be equal to the maximum overlap of the state 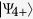 with a biseparable state. Since the set of biseparable states is convex and the state 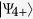 is invariant with respect to permutations of qubits, it suffices to maximize the overlap with pure biseparable states 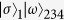 and 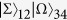. Here the subscripts label the four qubits, so for instance |*σ*〉_1_ is a single-qubit state and 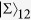 is a two-qubit state. We introduce explicit parametrization of these two states,


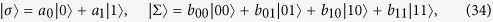


with the normalization conditions 
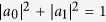
 and 
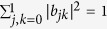
. It holds that


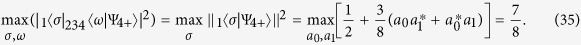


The maximization of 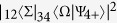 can be performed in a similar manner. We find that the optimal state 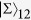 reads


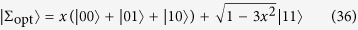


and the maximization of the overlap amounts to maximization of the function


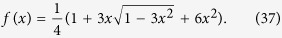


The maximum is achieved at 
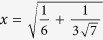
 and we arrive at


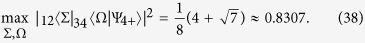


We thus find that 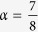 and the optimal projector witness for the state 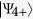 reads 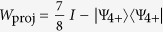.

## Additional Information

**How to cite this article**: Stárek, R. *et al*. Experimental investigation of a four-qubit linear-optical quantum logic circuit. *Sci. Rep.* **6**, 33475; doi: 10.1038/srep33475 (2016).

## Figures and Tables

**Table 1:** Properties of the three entanglement witnesses used to detect genuine multipartite entanglement of the four-qubit state 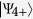.

	*W*_GHZ_	*W*_filter_	*W*_proj_
〈*W*〉	−0.112(2)	−0.0146(3)	−0.067(2)
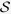	53	49	44
*p*_max_	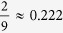	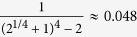	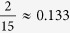

For each witness, the table displays its experimental mean value 〈*W*〉, the significance of the entanglement test 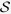 and the maximum tolerable fraction of white noise *p*_max_. For details, see text.

## References

[b1] Knill E., Laflamme R. & Milburn G. J. A scheme for efficient quantum computation with linear optics. Nature 409, 46–52 (2001). 11343107 10.1038/35051009

[b2] Kok P. . Linear optical quantum computing with photonic qubits. Rev. Mod. Phys. 79, 135–174 (2007).

[b3] Aspuru-Guzik A. & Walther P. Photonic quantum simulators. Nat. Phys. 8, 285–291 (2012).

[b4] O’Brien J. L., Pryde G. J., White A. G., Ralph T. C. & Branning D. Demonstration of an all-optical quantum controlled-not gate. Nature 426, 264–267 (2003). 14628045 10.1038/nature02054

[b5] Gasparoni S., Pan J.-W., Walther P., Rudolph T. & Zeilinger A. Realization of a photonic controlled-not gate sufficient for quantum computation. Phys. Rev. Lett. 93, 020504 (2004). 15323890 10.1103/PhysRevLett.93.020504

[b6] Zhao Z. . Experimental demonstration of a nondestructive controlled-not quantum gate for two independent photon qubits. Phys. Rev. Lett. 94, 030501 (2005). 15698243 10.1103/PhysRevLett.94.030501

[b7] Okamoto R., Hofmann H. F., Takeuchi S. & Sasaki K. Demonstration of an optical quantum controlled-not gate without path interference. Phys. Rev. Lett. 95, 210506 (2005). 16384126 10.1103/PhysRevLett.95.210506

[b8] Langford N. K. . Demonstration of a simple entangling optical gate and its use in bell-state analysis. Phys. Rev. Lett. 95, 210504 (2005). 16384124 10.1103/PhysRevLett.95.210504

[b9] Kiesel N., Schmid C., Weber U., Ursin R. & Weinfurter H. Linear optics controlled-phase gate made simple. Phys. Rev. Lett. 95, 210505 (2005). 16384125 10.1103/PhysRevLett.95.210505

[b10] Černoch A., Soubusta J., Bartůšková L., Dušek M. & Fiurášek J. Experimental realization of linear-optical partial swap gates. Phys. Rev. Lett. 100, 180501 (2008). 18518357 10.1103/PhysRevLett.100.180501

[b11] Gao W.-B. . Teleportation-based realization of an optical quantum two-qubit entangling gate. Proceedings of the National Academy of Sciences 107, 20869–20874 (2010). 10.1073/pnas.1005720107 PMC3000260 21098305

[b12] Lemr K. . Experimental implementation of the optimal linear-optical controlled phase gate. Phys. Rev. Lett. 106, 013602 (2011). 21231738 10.1103/PhysRevLett.106.013602

[b13] Zhou X.-Q. . Adding control to arbitrary unknown quantum operations. Nat. Commun. 2, 413 (2011). 21811242 10.1038/ncomms1392 PMC3267055

[b14] Lanyon B. P. . Simplifying quantum logic using higher-dimensional Hilbert spaces. Nat. Phys. 5, 134–140 (2009).

[b15] Mičuda M. . Efficient experimental estimation of fidelity of linear optical quantum Toffoli gate. Phys. Rev. Lett. 111, 160407 (2013). 24182241 10.1103/PhysRevLett.111.160407

[b16] Patel R. B., Ho J., Ferreyrol F., Ralph T. C. & Pryde G. J. A quantum Fredkin gate. Science Advances 2 (2016). 10.1126/sciadv.1501531 PMC4820377 27051868

[b17] Politi A., Cryan M. J., Rarity J. G., Yu S. & O’Brien J. L. Silica-on-silicon waveguide quantum circuits. Science 320, 646–649 (2008). 18369104 10.1126/science.1155441

[b18] Crespi A. . Integrated photonic quantum gates for polarization qubits. Nat. Commun. 2, 566 (2011). 22127062 10.1038/ncomms1570 PMC3482629

[b19] Metcalf B. J. . Quantum teleportation on a photonic chip. Nat. Photon. 8, 770–774 (2014).

[b20] Carolan J. . Universal linear optics. Science 349, 711–716 (2015). 26160375 10.1126/science.aab3642

[b21] Nielsen M. A. Optical quantum computation using cluster states. Phys. Rev. Lett. 93, 040503 (2004). 15323741 10.1103/PhysRevLett.93.040503

[b22] Walther P. . Experimental one-way quantum computing. Nature 434, 169–176 (2005). 15758991 10.1038/nature03347

[b23] Lu C.-Y. . Experimental entanglement of six photons in graph states. Nat. Phys. 3, 91–95 (2007).

[b24] Wieczorek W. . Experimental entanglement of a six-photon symmetric Dicke state. Phys. Rev. Lett. 103, 020504 (2009). 19659191 10.1103/PhysRevLett.103.020504

[b25] Yao X.-C. . Observation of eight-photon entanglement. Nat. Photon. 6, 225–228 (2012).

[b26] Wang X.-L. . Experimental ten-photon entanglement. *ArXiv e-prints* 1605.08547 (2016). 10.1103/PhysRevLett.117.210502 27911530

[b27] Eisaman M. D., Fan J., Migdall A. & Polyakov S. V. Invited review article: Single-photon sources and detectors. Review of Scientific Instruments 82 (2011). 10.1063/1.3610677 21806165

[b28] Lodahl P., Mahmoodian S. & Stobbe S. Interfacing single photons and single quantum dots with photonic nanostructures. Rev. Mod. Phys. 87, 347–400 (2015).

[b29] Schwartz I. . Deterministic generation of a cluster state of entangled photons. ArXiv e-prints 1606.07492 (2016). 10.1126/science.aah4758 27608669

[b30] Cerf N. J., Adami C. & Kwiat P. G. Optical simulation of quantum logic. Phys. Rev. A 57, R1477–R1480 (1998).

[b31] Barreiro J. T., Langford N. K., Peters N. A. & Kwiat P. G. Generation of hyperentangled photon pairs. Phys. Rev. Lett. 95, 260501 (2005). 16486324 10.1103/PhysRevLett.95.260501

[b32] Graham T. M., Bernstein H. J., Wei T.-C., Junge M. & Kwiat P. G. Superdense teleportation using hyperentangled photons. Nat. Commun. 6, 7185 (2015). 26018201 10.1038/ncomms8185 PMC4458874

[b33] Schreiber A. . Photons walking the line: A quantum walk with adjustable coin operations. Phys. Rev. Lett. 104, 050502 (2010). 20366754 10.1103/PhysRevLett.104.050502

[b34] Schreiber A. . A 2D quantum walk simulation of two-particle dynamics. Science 336, 55–58 (2012). 22403179 10.1126/science.1218448

[b35] Sansoni L. . Two-particle bosonic-fermionic quantum walk via integrated photonics. Phys. Rev. Lett. 108, 010502 (2012). 22304249 10.1103/PhysRevLett.108.010502

[b36] Hofmann H. F. Complementary classical fidelities as an efficient criterion for the evaluation of experimentally realized quantum operations. Phys. Rev. Lett. 94, 160504 (2005). 15904203 10.1103/PhysRevLett.94.160504

[b37] Flammia S. T. & Liu Y.-K. Direct fidelity estimation from few Pauli measurements. Phys. Rev. Lett. 106, 230501 (2011). 21770489 10.1103/PhysRevLett.106.230501

[b38] da Silva M. P., Landon-Cardinal O. & Poulin D. Practical characterization of quantum devices without tomography. Phys. Rev. Lett. 107, 210404 (2011). 22181862 10.1103/PhysRevLett.107.210404

[b39] Schumacher B. Sending entanglement through noisy quantum channels. Phys. Rev. A 54, 2614–2628 (1996). 9913770 10.1103/physreva.54.2614

[b40] Horodecki M., Horodecki P. & Horodecki R. General teleportation channel, singlet fraction, and quasidistillation. Phys. Rev. A 60, 1888–1898 (1999).

[b41] Jamiołkowski A. An effective method of investigation of positive maps on the set of positive definite operators. Reports on Mathematical Physics 5, 415–424 (1974).

[b42] Choi M.-D. Completely positive linear maps on complex matrices. Linear Algebra and its Applications 10, 285–290 (1975).

[b43] Hradil Z. Quantum-state estimation. Phys. Rev. A 55, R1561–R1564 (1997).

[b44] Ježek M., Fiurášek J. & Hradil Z. Quantum inference of states and processes. Phys. Rev. A 68, 012305 (2003).

[b45] Horodecki M., Horodecki P. & Horodecki R. Separability of mixed states: necessary and sufficient conditions. Physics Letters A 223, 1–8 (1996).

[b46] Terhal B. M. Bell inequalities and the separability criterion. Physics Letters A 271, 319- 326 (2000).

[b47] Lewenstein M., Kraus B., Cirac J. I. & Horodecki P. Optimization of entanglement witnesses. Phys. Rev. A 62, 052310 (2000).

[b48] Tóth G. & Gühne O. Detecting genuine multipartite entanglement with two local measurements. Phys. Rev. Lett. 94, 060501 (2005). 15783712 10.1103/PhysRevLett.94.060501

[b49] Jungnitsch B., Moroder T. & Gühne O. Entanglement witnesses for graph states: General theory and examples. Phys. Rev. A 84, 032310 (2011).

[b50] Jungnitsch B. . Increasing the statistical significance of entanglement detection in experiments. Phys. Rev. Lett. 104, 210401 (2010). 20867078 10.1103/PhysRevLett.104.210401

[b51] Steffen L., da Silva M. P., Fedorov A., Baur M. & Wallraff A. Experimental Monte Carlo quantum process certification. Phys. Rev. Lett. 108, 260506 (2012). 23004949 10.1103/PhysRevLett.108.260506

[b52] Mičuda M. . Tomographic characterization of a linear optical quantum Toffoli gate. Phys. Rev. A 92, 032312 (2015).

[b53] Gross D., Liu Y.-K., Flammia S. T., Becker S. & Eisert J. Quantum state tomography via compressed sensing. Phys. Rev. Lett. 105, 150401 (2010). 21230876 10.1103/PhysRevLett.105.150401

[b54] Shabani A. . Efficient measurement of quantum dynamics via compressive sensing. Phys. Rev. Lett. 106, 100401 (2011). 21469772 10.1103/PhysRevLett.106.100401

[b55] Emerson J. . Symmetrized characterization of noisy quantum processes. Science 317, 1893–1896 (2007). 17901327 10.1126/science.1145699

[b56] Moussa O., da Silva M. P., Ryan C. A. & Laflamme R. Practical experimental certification of computational quantum gates using a twirling procedure. Phys. Rev. Lett. 109, 070504 (2012). 23006350 10.1103/PhysRevLett.109.070504

[b57] Lu D. . Experimental estimation of average fidelity of a Clifford gate on a 7-qubit quantum processor. Phys. Rev. Lett. 114, 140505 (2015). 25910102 10.1103/PhysRevLett.114.140505

[b58] Hu X.-M. . Experimental creation of superposition of unknown photonic quantum states. *ArXiv e-prints* (2016) 1605.02339.

